# “She cannot use these methods before she has at least one child”: a qualitative study on family planning attitudes, knowledge, and experiences among adolescent girls and young women living with HIV in Moshi, Tanzania

**DOI:** 10.1186/s12978-026-02421-x

**Published:** 2026-08-04

**Authors:** Maeve F. Salm, Happyness Ngowi, Leonia Rugalabamu, Emanueli Msuya, Blandina T. Mmbaga, Joy Noel Baumgartner, Dorothy E. Dow

**Affiliations:** 1 Duke Center for Global Mental Health, Duke University, Durham, NC USA; 2 Duke Global Health Institute, Duke University, Durham, NC USA; 3 Kilimanjaro Christian Medical Centre, Moshi, Tanzania; 4 Moshi Youth Community Advisory Board, Moshi, Tanzania; 5 Kilimanjaro Christian Research Institute, Moshi, Tanzania; 6 Kilimanjaro Christian Medical Centre University, Moshi, Tanzania; 7 University of North Carolina-Chapel Hill, Chapel Hill, NC USA; 8 Department of Pediatrics, Duke University Medical Center, Durham, NC USA

**Keywords:** Family planning, Unintended pregnancy, Sociocultural norms, Reproductive choice, Adolescent girls and young women, HIV, Tanzania

## Abstract

**Background:**

Each year, about half of pregnancies in adolescents (15–19 years of age) are unintended, and the rate of adolescent pregnancy in sub-Saharan Africa is twice the global rate. Adolescent pregnancies have similar social determinants as those of HIV acquisition, resulting in adverse health outcomes for adolescent girls and young women (AGYW) and exacerbating adverse HIV-related health outcomes. To better understand reproductive choice for AGYW living with HIV (AGYWLWH), this study examined family planning attitudes, knowledge, and experiences among young women living with HIV in Moshi, Tanzania.

**Methods:**

Young female participants previously enrolled in the SYV pilot trial were purposively sampled to understand their attitudes, knowledge, and experiences with family planning services through in-depth interviews and a focus group discussion. Participants with a pregnancy experience (PE) and those without (NPE) were recruited to explore contextually relevant factors shaping these constructs. Thematic analysis included inductive and deductive coding, a data reduction matrix, and memo writing to identify and synthesize themes.

**Results:**

Of 16 enrolled participants, 13 had a pregnancy experience. The average age at first pregnancy was 20 years old, and almost two-thirds of participants experienced an unintended pregnancy (9/13). Participants shared that family planning services and hormonal contraception felt inaccessible until they had a pregnancy, often due to perceived social norms around sexual promiscuity or due to a fear of contraception-induced infertility. As a result, participants shared they did not feel well-informed about contraceptive options prior to their first pregnancy, while mentioning that contraceptive information pre-pregnancy often did not feel relevant. Therefore, no participants used hormonal contraception prior to their first pregnancy, and the most common method ever used was the male condom (8/16). After a first pregnancy, hormonal method use was more prevalent (9/13), and the most common hormonal method used was the implant (5/13). Almost all participants felt contraception was useful and promoted reproductive choice, citing the importance of birth spacing, particularly related to the successful prevention of maternal to child transmission. Despite perceived social norms related to family planning use pre-pregnancy, participants recommended AGYWLWH use family planning to achieve their personal and family composition goals, and they called for increased access to family planning information.

**Conclusion:**

A perceived social norm to have a child before family planning use emerged as the primary barrier to AGYWLWH reproductive choice, and almost all participants shared that family planning services were useful and promoted their autonomy. Looking to future generations, participants recommended increased access to contraceptive information for adolescents and young adults to improve reproductive choice for AGYWLWH.

**Supplementary Information:**

The online version contains supplementary material available at https://doi.org/10.1186/s12978-026-02421-x.

## Abstract (plain language summary)

Adolescent girls and young women living with HIV (AGYWLWH) face higher health risks from unplanned pregnancies. These young women often have limited access to family planning, increasing their pregnancy risk and reducing their reproductive choice. This study examined factors that influenced reproductive choice for AGYWLWH in Moshi, Tanzania through interviews and a focus group discussion. We found that participants believed family planning and hormonal contraceptive use was most appropriate for women who already had children. Many participants worried hormonal contraceptives caused infertility, and they perceived that AGYW seeking family planning services were labeled promiscuous. Despite these concerns, participants post-pregnancy expressed that contraception provided them with choice and freedom, and they shared preferences for individualized clinical counseling. Given their experiences with hormonal contraceptive use post-pregnancy, participants recommended increased access to contraceptive information for all adolescents and young people and encouraged family planning use for AGYWLWH. Based on these findings, we suggest implementing adolescent-responsive contraceptive services, comprehensive sex education in schools, and exploring family planning integration and evaluation in HIV clinics to increase reproductive choice for AGYWLWH.

**Supplementary Information:**

The online version contains supplementary material available at https://doi.org/10.1186/s12978-026-02421-x.

## Background

Approximately 21 million adolescents aged 15–19 years of age experience pregnancy annually in lower-middle income countries (LMICs), and about half of those (10 million) are unintended [1, 2]. In sub-Saharan Africa, the rate of unintended pregnancy is about three times greater than the rate in Europe and North America [2, 3]. Among women wanting to avoid a pregnancy in (LMICs), unmet contraceptive need is substantially higher for adolescents than for all women aged 15–49 (43% vs. 24%) [1, 4].

Adolescent girls and young woman (AGYW) aged 15–24 years also face higher risk of HIV acquisition compared to their male counterparts and experience unique health challenges. In 2023, about 4,000 AGYW acquired HIV every week, 77% of whom resided in sub-Saharan Africa [5]. AGYW newly diagnosed, as well as those living with vertically transmitted HIV who are pregnant, risk transmission of HIV to their infants [6]. Compared to adults, adolescents demonstrate less robust ARV adherence, are less likely to achieve viral suppression, and have greater viral rebound, often due to stigma experiences and challenges related to navigating sexual and romantic relationships [7, 8].

Pregnancy among adolescent girls and HIV acquisition are related to a myriad of overlapping inequities, many of which are structurally determined by socioeconomic, individual, and health service factors [1, 9, 10]. These structural factors can include poverty, gendered power dynamics, and access to education [11–13]. Adolescent pregnancies can further perpetuate cycles of poverty and structural violence, since they are often related to increased impoverishment, early marriages, and reduced educational attainment [1, 13]. Compounding these structural factors, AGYW in sub-Saharan Africa often experience stigma for having a pregnancy at this age (15–24 years) [14, 15]. This stigma can result in AGYW being refused from their homes and prevented from returning to school, leading to long-term psychological distress [14]. With an intergenerational impact, adolescent pregnancy can also exacerbate adverse maternal and child health outcomes, such as preterm birth, stunting, malnutrition, and eclampsia [1, 4, 9, 12, 16, 17] as well as adverse HIV health outcomes, such as poorer adherence to prevention of maternal to child transmission (PMTCT) recommendations and reduced antiretroviral (ARV) adherence [18]. The complex interplay of stressors and health outcomes between adolescent pregnancy and HIV management demonstrate the unique challenges facing adolescent girls and young women living with HIV (AGYWLWH).

With the 17th highest adolescent fertility rate in sub-Saharan Africa, 22% of adolescent girls in Tanzania (aged 15–19) gave birth in 2022, an increasing rate since 2010 [1, 19, 20]. Within Tanzania, the teenage childbearing rate ranges from 5% to 45% depending on the region, where teenage childbearing is defined as a first pregnancy experience or first birth when aged 15–19 [19, 20]. While teenage childbearing is related to a myriad socioeconomic and cultural factors, including individual choice, access to family planning services and contraception also influence adolescent pregnancy outcomes, particularly related to unintended pregnancy [21]. Within Tanzania, the contraceptive prevalence rate in 2022 among women 15–49 was 37% [3]. Approximately 40% of AGYW in Tanzania experience an unmet family planning need, defined as wanting to prevent pregnancy but not using contraception [10, 21]. When disaggregated by wealth quintile, approximately 40% of the wealthiest quintile experience an unmet need while about 60% of lowest wealth quintile experience an unmet family planning need [10]. In addition to these socioeconomic factors limiting access to contraception, Tanzanian policy from 2016 to 2021 also criticized the use of contraception and prevented pregnant AGYW from returning to school [22, 23]. More recently, the Tanzanian government has publicly committed to reducing adolescent pregnancies and reversed this policy, allowing AGYW to return to school two years after giving birth [24]. Despite the change in policy, AGYW with pregnancies continue to experience stigma, increasing adverse health outcomes and related health maintenance, particularly for adolescents who are living with HIV and pregnancy [23].

Given the varied stressors facing young people living with HIV (YPLWH), a mental health and life skills intervention, called Sauti ya Vijana (SYV), was developed with and for YPLWH to improve mental health and HIV outcomes [25]. From the pilot study with longitudinal follow-up data to 30-months in Kilimanjaro, Tanzania, it was noted that at least 14% (8 of 56) of AGYW who were randomized in the pilot became pregnant, had a child, and were subsequently lost to follow-up [25], more than double the teenage childbearing rate in the Kilimanjaro Region (6%) [20]. This qualitative sub-study was designed to better understand reproductive choice among AGYWLWH by exploring family planning attitudes, knowledge, and experiences among female participants enrolled in the original SYV study.

## Methods

### Setting

This qualitative sub-study occurred in Moshi, Tanzania from July to December 2022. Located within the Kilimanjaro region, Moshi is a peri-urban environment, home to the Kilimanjaro Christian Medical Center (KCMC), a zonal referral hospital which provides high-quality, specialized care to about 11 million people [26]. Moshi includes 12 additional hospitals, such as the Mawenzi Regional Hospital and the Majengo Health Center [27, 28]. Perceived as a neutral facility by participants living with HIV, all research activities related to the former SYV pilot and this qualitative sub-study occurred at the Majengo Health Center in private rooms.

### Participants

Participants (*n* = 68 adolescent girls) were recruited from the NIH-funded SYV pilot trial cohort [25]. SYV participants enrolled in the pilot study were originally recruited between 2016 and 2017 through the KCMC or Mawenzi HIV youth clinics (Teen Club, a monthly group education program offered during routine HIV care for YLWH) [25]. Inclusion criteria for the original SYV pilot study were: (1) youth between the ages 12–24 (average age was 17 years), (2) attended Teen Club for YPLWH at KCMC or Mawenzi, (3) received ARVs, (4) understood and were disclosed to their HIV diagnosis, and (5) provided written informed consent (or assent with caregiver consent if under 18) [25]. Inclusion criteria for this qualitative sub-study were female participants from the SYV pilot study who were age 18 years or older, were reachable, and still resided near Moshi, Tanzania to attend in-person study activities.

Recruitment for the qualitative sub-study was led by a local research assistant involved in the SYV pilot study (HN). Female participants enrolled in the SYV pilot study cohort were purposively recruited and contacted by phone to determine eligibility. Participants were called at least three times to determine reachability. Participants who moved out of Northeastern Tanzania or were unreachable were ineligible for the study. To explore reproductive choice based on contextually relevant factors, participants who had pregnancy experiences (PE) and those who did not have pregnancy experiences (NPE) were recruited. Eligible individuals who were reached were invited to an informational study meeting at the Majengo Health Center where they learned about all study activities and consented and enrolled as desired. Interested individuals provided written informed consent in Swahili in a private room. Participants who were unable to attend the introductory meeting, but who expressed interest by phone, consented to the study on the day of their in-depth interview (IDI). To capture both personal perspectives and insight on community norms, all IDI participants were invited to participate in a focus group discussion (FGD). When referencing participants in this sub-study, participants will be quoted with their age, age at first pregnancy (indicated as "P1"), or NPE accordingly.

### Data collection instruments

Study activities included in-depth interviews (IDIs) and a focus group discussion (FGD). IDIs provided personal insight into family planning knowledge, attitudes, and experiences, while the FGD elicited community norms about perceived appropriate use of family planning and recommendations for future support to AGYWLWH in achieving reproductive choice [29]. IDI and FGD guides were informed by literature on family planning use and access [30, 31] and input from local experts and community members. Tanzanian members of the research team (HN, LR) and a community advisory board member (EM) helped further refine the guides to improve the cultural relevance and align with important topics for youth related to sexual and reproductive health (Supplemental Materials 1–3). These guides were translated into Swahili and back translated into English, and they were pre-tested by the interviewer (HN).

During piloting of the guide, it was noted that “family planning,” defined by the World Health Organization as the ability of individuals to achieve their desired family size, birth timing, and spacing via contraceptive methods and infertility treatment [32], and “contraceptives” meaning the specific behaviors or technologies used to prevent pregnancy [33], were being used interchangeably in Swahili as “uzazi wa mpango,” which directly translates to “reproductive plan.” While the Swahili term for contraceptives, “vidhibiti mimba,” is used clinically, a community advisory board member indicated that this term was not widely known outside of the medical community and therefore was not recommended. As a result, the team (MS, HN, EM, LR) clarified these terms to reflect the family planning components of interest. These components of interest included: family planning advice or counselling (“ushauri ya uzazi wa mpango”), clinical family planning services (“huduma za uzazi wa mpango”), and family planning methods i.e. contraceptive methods (“njia za uzazi wa mpango”). The following contraceptives were included in the interview guides: male condom, calendar method, oral contraceptive pill, implant, contraceptive injection, and intrauterine device (IUD or “the loop”). When referring to hormonal contraception throughout the paper, this includes the pill, implant, injection, and IUD.

### Data collection procedures

The research assistant (HN) was trained in qualitative interviewing and received a refresher training specific to this study. All IDIs and the FDG were audio recorded, and they were facilitated by the research assistant (HN) with semi-structured guides in Swahili at the Majengo Health Center in private rooms. Childcare was provided for participants with children to support their participation and allow for a private discussion. Participants were then reimbursed for their transport and compensated for their time. All IDIs and the FGD were transcribed and translated from Swahili into English by one translator. Data were reviewed by the investigator (DD) and interviewer (HN) for accuracy. IDIs lasted from 45 to 75 min, and the FGD (*n* = 6 PE participants) lasted for about 120 min.

### Analysis

Applied thematic analysis was used to analyze the data because it is a flexible strategy that allows for the identification of emergent themes, an analytic method that could support a data-driven exploration of attitudes, knowledge, and experiences around reproductive choice [29, 34]. NVivo version 12 was used to code transcripts [35]. Codes were deductively and inductively constructed by the primary author (MS). A deductive approach was first used to understand a priori themes of interest (e.g. attitudes toward contraception, sources of family planning knowledge, experiences with family planning). Deductive coding was literature-driven, based on sections of the interview guide, and led to the structural codes. An inductive approach was then used to center participant voices in the analysis process. Inductive codes were developed by examining emergent themes that arose over five PE transcripts, which were refined and modified based on another five PE transcripts. This preliminary codebook was applied to the remainder of the transcripts. No further themes emerged from the PE or NPE transcripts, so no further codes were added to the codebook. The primary author (MS) then coded all transcripts with the finalized codebook (Supplemental Material 4). Given the codebook refinement process and lack of additional emergent themes from NPE interviews, it was determined that saturation was reached [36].

In conjunction with the coding process, the primary author (MS) wrote reflection and document memos to support the conceptualization and understanding of participants’ responses. Coding reports for each code were printed by the primary author (MS) to summarize main points, identify nuances and disagreements, and consider further themes and questions. Following this, analytic or cross-code memos were composed by the primary author (MS) to explore participants’ attitudes, knowledge, and experiences with family planning. These analytic memos facilitated the synthesis of ideas expressed in the transcripts, the identification of poignant quotes, and reflection on researcher biases or assumptions. Given the positionality of the primary author (MS)—a U.S. resident, predominantly trained in Western biomedical epistemologies—, the primary raw coding reports were shared with Tanzanian research assistants (HN, LR) to reduce bias related to positionality. The Tanzanian research assistants (HN, LR) provided their perspectives on major themes that emerged from the coding reports and confirmed the thematic results synthesized in the analytic memos and articulated below. Additionally, a data reduction table was compiled to synthesize quantifiable information across participants.

## Results

### Participant characteristics

Of the 68 female participants enrolled in the original SYV pilot study, individuals who were not reachable or were unavailable (*N* = 52) were ineligible for the study (Supplemental Material 5). Sixteen young women consented to participate, 13 who had pregnancy experiences (PE), two of whom were currently pregnant, and three participants who did not have a pregnancy experience (NPE). Based on participant demographics at baseline enrollment in the SYV pilot study (2016–2017), there were no differences between female participants enrolled in this qualitative sub-study (*n* = 16) compared to those not enrolled (*N* = 52), except this sub-study sample experienced HIV disclosure at an older age (*p* = 0.032, Supplemental Material 6). One participant in the sub-study acquired HIV behaviorally. Only PE participants joined for an FDG; NPE participants were not available to participate in multiple study activities.

Of PE participants, the majority (11/13) had their first pregnancy experience between 18 and 22 years of age (Table 1). All living children (*n* = 11) born to these AGYWLWH were HIV exposed and uninfected at the time of interview. One participant (1/16) was married at the time of pregnancy and remained married at the time of the interview.

**Table 1:** Participant characteristics*

	PE (*n* = 13)	NPE (*n* = 3)
Age**	23	21
Age at first pregnancy** (range)	20 (16–24)	--
Total pregnancies	17	--
First pregnancy	13	
Second pregnancy	4	--
Unintended first pregnancy	7	--
Unintended second pregnancy (*N* = 4)	2	--
Adolescent pregnancy (15–19 years)^+^	4	--

Dashed lines (--) indicate non-applicable information, since NPE participants did not experience pregnancy

*This table reflects participant characteristics at the time of the interview. **Mean value in years. ^+^Adolescent pregnancy as defined by the UNFPA [1, 20]

### Thematic results

Participant responses about family planning are organized into the primary constructs of the interview guide: attitudes and social norms, knowledge, experiences, and recommendations for AGYWLWH. Throughout the data, salient differences emerged within these constructs pre-and post-pregnancy related to hormonal and non-hormonal contraceptive methods. NPE participants were pre-pregnancy at the time of interview, and their perspectives were consistent with PE perspectives pre-pregnancy.

### Personal attitudes and perceived social norms for family planning

Participants frequently referenced perceived social norms that limited engagement with family planning, including access to clinical services and contraceptive use. Young women shared that family planning felt inaccessible until they had a child or attended services with a partner, otherwise they felt AGYW were perceived as sexually promiscuous. Participants also worried that hormonal contraception could impact their fertility or reproductive health.

#### Perceived appropriateness: “because I didn’t have a child”

All participants commented about the perceived relationship between childbearing and engagement with family planning services. Specifically, participants felt family planning services were reserved for *“people who have families” (24 years old*,* 22 P1).* When asked why a participant had not used hormonal contraception previously, she replied, *“Because I didn’t have a child*,* therefore I never thought to follow family planning” (25 years old*,* 20 P1)*. These perceptions shaped their thoughts on appropriate family planning use. *“As I know it*,* family planning is used after childbirth” (22 years old*,* 22 P1)*, and *“I wanted [hormonal contraception] at that time because I already had a little child” (25 years old*,* 20 P1)*. Another participant mentioned that *“…I think that women who have children are the main target [of family planning services]*,* and I see that they go with their husbands” (21 years old*,* NPE).*

Many participants suggested that a fear of side effects or inability to conceive drove their preference for having a child before hormonal contraceptive use. *“I used to hear people saying that if you install these things and you are still a girl*,* it becomes harmful. It requires grown-up women who have children” (22 years old*,* 21 P1).* Concerns about infertility were often intertwined with age, which were expressed during interviews and during the FGD as well, with a participant saying,

*“People want to know if they have a problem in the womb and that is why they wait until they get pregnant. Once she has given birth*,* that is when she starts looking for the methods. She cannot use these methods before she has at least one child. Also*,* the community does not have much education concerning family planning. They are told that family planning methods are harmful. It is possible for you to use it and fail to get pregnant completely. It might damage the womb or things like that*,* and this causes fear to the people” (FGD).*

Conceiving prior to hormonal contraceptive use seemed to be a widely held norm,

“*About family planning*,* they say that a woman with children is the one who can receive them. It is not written anywhere that a girl that has no children should be using them (they laugh)” (FGD).*

#### Perceived side effects: “I am afraid of it personally”

All participants expressed concerns about the side effects of hormonal contraception, and that these concerns hindered method use. One participant mentioned apprehension around use,

*“Because my friend would tell me…if it likes you*,* you gain weight and if it refuses you*,* you lose weight*,* and your bleeding becomes longer. So that is why I am afraid of it personally” (24 years old*,* 22 P1).*

Beyond changes in body habitus and disruption of the menstrual cycle, there were also ubiquitous concerns about infertility. One participant shared, *“I think with young people it is not good to use family planning methods because they are still young—there must be side effects” (19 years old*,* NPE).* Other perceived side effects that participants cited included device movement, cancer onset, swelling, and uterine and fallopian tube damage.

#### Partner presence: “I am not prepared to use them…I don’t live with a husband yet”

Similarly, participants expressed how romantic partnerships influenced perceived access to family planning services. A few participants implied that family planning services required both individuals in the relationship to access clinical services, which in some cases could prevent use.

*“My opinion is that with these small girls*,* when they come to the dispensary without their spouses—they are supposed to come with their spouses—you find they are students*,* or they were deceived [by their partner]” (24 years old*,* NPE).*

This same participant was also *“…advised that I should be using [contraception] with my lover” (24 years old*,* NPE)*, so she decided not to use family planning services until she had a long-term partner. Another participant mentioned how information about hormonal contraception was less relevant to her without a spouse. *“The information influenced me*,* but I am not prepared to use [contraceptives] because I don’t live with a husband yet” (24 years old*,* 24 P1).*

#### Perceived social stigma: “they might wonder if I am a sex worker”

Participants expressed that using family planning services before pregnancy or romantic relationships was associated with sexual promiscuity. When asked why she never sought family planning services, one participant replied,

*“I think that going to someone to ask about family planning is a little awkward because I am worried ‘what are they going to think about me?’ They might wonder if I am a sex worker or something like that” (21 years old*,* NPE).*

Another participant shared a similar reflection, demonstrating how it impacted the perceived relevance of contraception. When asked why she had not used hormonal contraception before pregnancy, she replied,

“*Because if you use [hormonal contraception]*,* then people might ask you questions like*,* ‘why are you using it? You are not married yet.’ Therefore*,* I used to think that they were useless to me at that time…” (22 years old*,* 19 P1).*

This sentiment was echoed by many other participants, including during the FGD when a participant mentioned, *“…if you are in school and people know that you use the [implant]*,* they would say you are a sex worker*,* and that is why you are afraid that you might get pregnant” (FGD).*

### Knowledge: pre-pregnancy

Personal attitudes and perceived social norms shaped participant relationships with family planning knowledge pre-pregnancy. As a result, these norms often limited access to clinical information and reduced the perceived relevance of contraceptive information until a first pregnancy.

#### Unfamiliarity: “I didn’t know any other [method] to use”

Most participants shared that their lack of knowledge about contraceptive options was a significant reason why they did not use contraception, either prior to their first pregnancy or currently. When asked why she had not wanted family planning prior to her first pregnancy, a participant responded, *“Because I didn’t know” (25 years old*,* 20 P1).* Another participant had not used hormonal contraception before her first pregnancy, *“…because I still hadn’t received the family planning education. Therefore*,* I didn’t have any intention to use family planning methods” (24 years old*,* 16 P1).* If participants used non-hormonal methods pre-pregnancy, they mentioned how a lack of knowledge reduced their ability to choose which contraceptive method they felt was most appropriate for them. When asked why she decided to use a condom, a participant commented, *“… because I didn’t know any other [method] to use” (23 years old*,* 19 P1).*

#### Relevance of information: “I was still a girl…I didn’t consider such matters important”

While participants felt they had not received sufficient family planning information pre-pregnancy, most participants shared that family planning information was not relevant to them prior to pregnancy. When asked how contraception information influenced her use before pregnancy, one participant mentioned, *“It didn’t influence me because I was still a girl*,* and I didn’t consider such matters important” (22 years old*,* 21 P1).* Another participant had an unintended pregnancy at 16 years of age and sought an abortion. When asked why she had not used contraception, pursued family planning services, or talked to a healthcare provider about family planning prior to her first pregnancy, she responded, *“I never needed to use it. I didn’t even have such thoughts” (24 years old*,* 16 P1).* These sentiments were echoed by other NPE participants as well, sharing, *“There was no important information to me (she laughs) because right now*,* I don’t have such intentions” (19 years old*,* NPE)*, and *“I didn’t follow [family planning information] up too deeply…because the way I heard them speaking*,* I just considered them matters of the adults (she laughs)” (21 years old*,* NPE).*

### Knowledge: at the time of interview

After sharing their former knowledge about family planning and contraceptives pre-pregnancy, participants reflected on sources of family planning information over their lifetime, which contraceptive methods they were aware of at the time of interview, and possible reasons for using contraception.

#### Sources of information: “my mother told me…”

Across participants’ lifetimes, knowledge of family planning was primarily obtained from close relationships, such as mothers, partners, friends, or other relatives, such as sisters and aunts.

*“[My mother] told me*,* ‘I understand that you have grown up and you also have your own desires. When you think that you are overwhelmed*,* it is better that you use family planning…” (19 years old*,* NPE).*

Participants also heard about family planning from people in their social network, such as neighbors (*n* = 2) or *“people around me”* (*n* = 6). Outside of their social networks, participants received information from the clinic, public service announcements, and school (Table 2).

**Table 2:** Sources of contraceptive information

Sources of Information	*N* = 16
Close relationships	15
Mother	7
Partner	5
Friends	5
Other relatives	5
Other social networks	8
Clinical sources	6
Public communications	5
School	3

Few participants sought information from clinical sources prior to pregnancy, and most participants who received clinically sourced information about family planning did so after pregnancy (*n* = 5). Of those who first learned about family planning from a clinical source, two participants learned about contraception at delivery, one participant learned from a seminar, and three others mentioned a general clinical source.

#### Contraceptive knowledge: awareness and “protection”

Most participants were aware of several contraceptive methods at the time of interview, including oral contraceptive pills (*n* = 12), male condoms (*n* = 12), and female condoms (*n* = 7). Despite method awareness, participants often asked the interviewer questions about proper use and related side effects.

When discussing the purpose of family planning, all participants mentioned *“protection.”* Some mentioned that contraception could *“protect”* from unintended pregnancy while others highlighted that contraception could facilitate recommended birth spacing. For example,

*“In giving birth*,* you should use family planning because you should put some space between the children*,* so the children can be healthy and strong and you can rear the children well…” (22 years old*,* 22 P1).*

Two participants explicitly mentioned that a lack of sufficient space between births could increase the risk of maternal to child transmission of HIV, saying,

*“…if someone like myself [living with HIV] has children in quick succession*,* the child will get the infection. But if you follow the instructions from the doctors*,* leaving intervals between the children*,* the children will be safe” (22 years old*,* 19 P1).*

Most participants also mentioned protecting themselves or their partners from the transmission or acquisition of STIs or HIV through condom use, *“…if you use family planning such as condoms*,* you could protect yourself from diseases” (24 years old*,* 19 P1)*, and *“In our condition you have to use [the condom] so that you can protect your partner from harm” (22 years old*,* 21 P1)*.

### Participant experiences with family planning

Perceived social norms about family planning and the resulting knowledge about contraception shaped hormonal method and service use pre-pregnancy, impacting intended pregnancy outcomes. After participants had a pregnancy, perceived access to contraception and family planning services shifted, and participants offered perspectives about preferred contraceptive characteristics and family planning counseling. Amidst the complexity of norms, knowledge, and experiences shaping family planning behaviors, romantic partner encouragement or disapproval of hormonal contraception also influenced contraceptive use.

#### Lifetime contraceptive use

At the time of interview, 10/16 participants were using any type of contraception, and two participants had never used contraception (Table 3). Among all participants, the most common contraceptive method was the male condom (8/16, 50%). No participants used a hormonal method of contraception prior to pregnancy. After a first pregnancy, hormonal methods were commonly used (9/13, 69%), the most common being the implant (5/13, 31%). Of the 14 participants who had ever used contraception, they tried multiple different methods to determine the best fit for them (range: 1–4 different method types, average: about 2 different types).

**Table 3:** Lifetime experience with contraceptive methods

Method ever used	PE	NPE	Total (*N* = 16)
Never used a method	1	1	2 (13%)
Non-Hormonal Method			
Male condom	6	2	8 (50%)
Calendar method	6	1	7 (44%)
Other (Male pulls out or “The Ashes”)	2	0	2 (13%)
Hormonal Method			
Oral Contraceptive pill	2	0	2 (13%)
Intrauterine device (IUD, “the loop”)	3	0	3 (19%)
Implant (“Norplant”)	5	0	5 (31%)
Injection	2	0	2 (13%)

#### Pre-pregnancy method use and related pregnancy outcomes

Of the 13 PE participants, there were 17 pregnancy experiences. Nine pregnancies were unintended (7/13 first pregnancies, 2/4 second pregnancies) across different participants, resulting in 9/13 individuals experiencing unintended pregnancy. One of the seven unintended first pregnancies ended in an unsafe abortion. Of the six PE participants who had an intended pregnancy, three participants used non-hormonal contraception prior to pregnancy. Of those who had an unintended pregnancy, three used a non-hormonal contraceptive method prior, one of whom said that she had stopped using contraception (a condom) and then experienced the unintended pregnancy (24 years old, 16 P1). The other participant mentioned that she used the calendar method, and *“…the calendar method let me down because I fell pregnant” (23 years old*,* 20 P1)*. The third participant did not discuss her use of contraception in relationship to her unintended pregnancy, but she used the calendar method prior to pregnancy (24 years old, 22 P1).

#### Preferred contraceptive characteristics: easy to use, long-acting, and limited side effects

Many participants expressed that ease of use and ease of removal were preferrable contraceptive characteristics. For example, one participant said, *“I preferred [Norplant] because I felt that it was the easier way for me…that one is of the arm*,* unlike the loop which is challenging” (22 years old*,* 21 P1)*. Ease of use was expressed by another participant who discussed her preference for the calendar method.

*“I learned and understood the calendar well*,* and my husband also understands it a little bit. Then I thought that I shouldn’t be running around looking for [hormonal] contraception while this calendar method is useful to me” (25 years old*,* 20 P1).*

Participants also expressed preference for long-acting contraceptives and contraceptives without side effects. When discussing why she preferred the implant, one participant mentioned it was *“because it was a long-time method that I could use to prevent me from getting another child” (24 years old*,* 19 P1)*, highlighting the length of coverage as a desired characteristic. Another participant commented that she preferred the implant because, *“It has not harmed me nor given me trouble in any way. Therefore*,* it is the best method for me” (24 years old*,* 16 P1)*.

#### Counseling preferences: individual, clinical, and familiar

Given concerns about side effects and the variability in contraceptive experiences, some participants highlighted the importance of individualized counseling to better understand *“whether it will accept me.”* This sentiment about the variability and individuality of experience was echoed throughout interviews, even shaping whether participants would recommend hormonal contraception to their friends. For example, a few participants did not want to recommend hormonal contraception because, *“…some people say that they depend on whether they like your body or not. Therefore*,* you never know if your friend uses it*,* whether it will agree with her or not…” (25 years old*,* 20 P1).* During the FGD, another participant mentioned that *“Each individual has her own way of thinking about the methods that are safe to her” (FGD).*

With this emphasis on individual experiences, participants often expressed a preference for receiving contraceptive information from clinicians.

*“I think that the person I could discuss family planning with is my mother and perhaps the doctor…because if my mother can advise me*,* the doctor can advise me because he knows more” (24 years old*,* 16 P1)*.

As a result, a few participants shared that the clinical information they received facilitated hormonal contraceptive use. For example, one participant stated, *“After being given the [clinical] family planning education*,* that is when I decided to use [hormonal contraception]” (23 years old*,* 20 P1).* Another participant described that her provider was the most influential source of information, facilitating contraceptive choice. *“The doctor asked me about what kind of family planning I was using*,* told me that there were several different ways*,* and I chose the loop” (24 years old*,* 16 P1).* Participants felt strongly about clinical education as a facilitator to contraceptive use, even recommending it be a prerequisite to use. *“[AGYWLWH] should first be given education before using [hormonal contraception]” (25 years old*,* 20 P1).*

Beyond a preference for clinically-sourced information, several participants preferred to receive their information and services from clinics where they had trusted relationships. For example, when asked why she sought family planning services from a local hospital, she replied she felt *“free”* to ask about family planning there *“because it has been my clinic since I was little” (21 years old*,* 20 P1).* A similar sentiment was expressed by NPE participants. *“I would like to go to [local hospital] because there are some doctors that I am accustomed to. Therefore*,* I won’t be afraid to ask them questions…I will be free” (19 years old*,* NPE).* Other participants mentioned they received or sought family planning services from the hospital where their child received care. Two participants asked about contraception at their HIV Care and Treatment Center (CTC) *“…because it is an area that I know well and I am accustomed to” (24 years old*,* 19 P1).* When other participants were asked why they did not receive contraceptive information from their CTC, common responses were *“I never thought about it” (23 years old*,* 19 P1)* or *“because I haven’t reached that stage yet – the state of having children” (24 years old*,* NPE).*

#### Coercive clinical experiences: “they put it in for me…I didn’t want it…”

While most participants had positive experiences when receiving tailored family planning counseling at a trusted clinic, two participants had coercive experiences. One participant mentioned that she did not have the option to discuss different contraceptives or ask questions about the method (IUD) she received before it was inserted under anesthesia post-Cesarean Section. Another participant experienced coerced insertion when she attempted to leave her child’s HIV check-up, but she was not permitted to leave with the required documentation until she consented to contraception (IUD).

*“…they held our clinic cards and said*,* ‘You will not leave until we measure your reproductive path [uterine length]*,* and things like that for cervical cancer.’ Therefore*,* they measured it. They asked me what method of family planning I was using*,* and I told them that I wasn’t using any method. That is when they put [the IUD] in for me…I didn’t want it…I didn’t even know that family planning existed” (25 years old*,* 20 P1).*

This participant eventually removed the IUD due to the pain it caused, which impacted her ability to engage in sexual activity with her partner.

#### Partner encouragement or disapproval

Participants frequently mentioned how their romantic partners’ support or disapproval of contraception influenced their use of desired methods. For example, one participant felt encouraged that her partner supported hormonal contraceptive use, sharing,

*“He says ‘you should [use the implant] because having a child before the other one is grown enough is not good. The other child will not be healthy. And you need to be healthy enough yourself to be able to carry another pregnancy’” (25 years old*,* 20 P1).*

Another participant shared that her partner obtained contraception for them, *“He just told me that he had brought [a condom] with him and therefore I shouldn’t worry…I felt good because very few men do that” (23 years old*,* 19 P1).*

That said, a few participants expressed that their partners discouraged hormonal contraceptive use, often citing perceived side effects as the rationale for resistance. *“I told him that I had put this [implant] in me. He was opposed to it*,* asking me*,* ‘Why did you put it in you? Don’t you know the side effects of this?’” (22 years old*,* 19 P1).* Partner opposition to hormonal contraceptive use was also a prevalent theme in the FGD. Participants said romantic partners may react with, *“You are putting that [contraceptive unspecified] one in?! Do you know the consequences? You could get cancer” (FGD)* or *“You can get a swelling. Do you know if you will get children or not? Why are you using such things?” (FGD).*

### Looking to the future: family planning recommendations for AGYWLWH

There was almost universal agreement among both PE and NPE participants that family planning was important and useful, resulting in recommendations that AGYWLWH explore family planning options to help them achieve their goals. To support AGYWLWH in this process, participants strongly recommended increased access to contraceptive information for this population, their partners, and the community.

#### Utility of family planning: choice, freedom, and “reaching objectives”

Almost all participants perceived family planning as a tool to support their choice in pregnancy, family composition, and personal “objectives” or life goals, with several participants describing contraception as freeing. For example, one participant mentioned, *“…family planning has helped me. You could get many children*,* but if you have planning it helps.” (23 years old*,* 19 P1).* Beyond personal experience, another participant shared, *“My opinion of family planning is that people need to know how it is used and that it is useful*,* so that they can plan the arrangement of their children” (21 years old*,* NPE).* During the FGD, one participant commented that AGYWLWH should *“…[use contraception] to reach their objectives” (FGD).* As a result, many participants recommended family planning services for other AGYWLWH. One participant said AGYWLWH should use hormonal contraception, *“Due to what happened to me before using it…I wouldn’t like to see another person’s dreams evaporating before they were accomplished” (23 years old*,* 20 P1).*

#### Desired education: “let’s not hide these matters from [young people]”

All participants called for increased education about family planning and contraception, both to address personal needs and to increase the accessibility of contraceptive information for young women, young men, and the community at large. When asked why AGYW do not use family planning services in the FGD, one participant responded,

*“It is because they don’t have enough education concerning family planning. If only they knew that these methods are aimed at protecting them from getting pregnant unexpectedly*,* there wouldn’t be such thing as saying*,* ‘If you use these methods*,* you will not get children’” (FGD).*

When asked how she would improve family planning services, another participant responded,

*“I would recommend subjects in schools because that is where things begin. Let’s not hide these matters from [young people]. We should tell them everything about family planning; methods to be used that are good for them and are free of problems” (22 years old*,* 20 P1).*

Improved access to education was the most salient theme from the FGD. When asked about potential strategies to support AGYW use of family planning services, one participant mentioned,

*“I would give them education*,* including to those who do not yet have children*,* so that they would be educated properly about the use of family planning. They shouldn’t just follow what is said in our community…so she can reach her objectives and have a child at the right time for her” (FGD).*

## Discussion

Personal attitudes and social norms limited access to family planning services for AGYWLWH in Moshi, Tanzania. Participants perceived family planning services and hormonal contraception as most appropriate for adults who already had a pregnancy. These perceived social norms prevented participants from accessing clinical family planning services before their first pregnancy, reducing their access to contraceptive information and therefore reproductive choice. As a result, the primary source of contraceptive information for AGYWLWH was close relationships, so incomplete information, and sometimes misinformation, about hormonal contraception was prevalent pre-pregnancy. Rather than risk infertility, a common misconception about hormonal contraceptive use, AGYWLWH preferred using non-hormonal methods of contraception prior to their first pregnancy, contraceptive options which are most prone to method failure and subject to partner approval [3, 37, 38]. Amidst this complex intersection of social norms, limited access to contraceptive information, and fear of side effects, 7/13 participants experienced an unintended first pregnancy.

Participants who had a child felt family planning was accessible and appropriate post-pregnancy, so they were able to and preferred to receive information from providers about hormonal contraceptive options. Participants who used hormonal contraception felt it was useful to their health, family composition goals, and professional goals. Despite the perceived increase in hormonal contraception accessibility post-pregnancy, half of the second pregnancies (2/4) reported were unintended. Post-partum care recommendations emphasize access to desired contraception [39], and these results encourage a continued exploration of desired post-partum family planning access.

Within this sub-study, the cultural norm to have a child before attending family planning services emerged from fears that hormonal contraception caused infertility and was associated with sexual promiscuity. Similar fears of side effects have been reported by adolescents across Tanzania, East Africa, and other LMICs [1, 40–47]. Within Tanzania and other LMICs, concerns about contraceptive-induced infertility have been shared by healthcare providers, hindering family planning provision for adolescents [40, 46, 47]. Echoing perceptions of AGYWLWH in this study, contraceptive use among AGYW has also been associated with sexual promiscuity [1], resulting in internalized stigma and stigmatizing comments from partners [40, 43, 48, 49], providers when pursuing hormonal contraceptive methods [40, 46, 47], and the general community. Participants implied that pursuing family planning services with a romantic partner can reduce some experiences of stigma, sentiments shared by AGYW in other countries [48]. The combined fear of infertility and stigma related to sexual promiscuity in this context culminated in a widely held norm that AGYWLWH should wait to access clinical family planning services and use hormonal contraception until after their first child. As a result, hormonal contraceptive information felt irrelevant to participants pre-pregnancy. The perception that family planning and hormonal contraceptive use is not appropriate for AGYW has also been observed in other regions of Tanzania, citing similar sentiments related to romantic partner influences and misconceptions about contraception [50]. Against the backdrop of former Tanzanian policies, AGYW may perceive family planning as services for “women with children” due to an inability to return to school [23, 24]. While these policies were revoked in 2021 [24], further investigation into the impact of political factors on family planning access may help elucidate additional barriers for AGYW.

While these sociocultural norms about family planning use likely shape health behaviors for all AGYW in the Moshi community, it is possible these cultural perspectives have a larger impact on AGYWLHW. Former studies with AGYWLWH elucidated that they may believe they cannot conceive due to living with HIV [51]. Given that almost all participants in this study acquired HIV by vertical transmission, it is possible there was greater uncertainty about conceiving prior to family planning use in this population, potentially inflating concerns about infertility related to hormonal contraceptive use. With an increasing number of AGYW with vertical HIV transmission transitioning to adulthood, it is critical to affirm they can have children and have children born HIV-free.

Despite participant reports that contraceptive information lacked relevancy pre-pregnancy, participants recommended education be provided to AGYW to improve family planning outcomes. Navigating this tension will require innovative strategies that transform community norms, address misinformation, and appeal to AGYW. Community-engaged sensitization efforts could address cultural norms about appropriate family planning users and misinformation about hormonal contraceptive side effects [52–55]. Since these norms and misinformation were expressed by all participants, strategic engagement of caregivers, community leaders, and other local champions in sensitization campaigns is recommended [52–55]. The youth and adult community advisory boards (CAB) in Moshi have a strong history of galvanizing impactful sensitization campaigns about HIV stigma, and they could facilitate conversations about family planning engagement and adolescent sexual and reproductive health. Peer education and referral models have also successfully increased access to and normalization of family planning use among young people, a model that could be adapted by Moshi’s youth CAB [52, 53, 56, 57].

Additionally, participants mentioned that romantic partner support or disapproval of hormonal contraception influenced use, a phenomenon observed in multiple settings [40, 43, 47, 48, 58]. Increasingly, interventions have encouraged both partners attend family planning services to improve young men’s understanding of contraception [55, 56]. With participants expressing a perceived pressure to access family planning services with a romantic partner, partners’ influence on method use, and misconceptions expressed by partners, this study demonstrated a need to for more robust and innovative engagement of adolescent boys in family planning education. School-based comprehensive sexual education (CSE) should be provided to all adolescents and young adults, with an emphasis on a safe and participatory learning environment, gender and power influences on family planning, and evidence-based information about contraception [56, 57, 59–61].

To increase the perceived relevance of contraceptive information pre-pregnancy, some interventions have employed targeted messaging approaches to match the desires of Tanzanian adolescents [55]. Participants suggested that increased messaging about “reach(ing) objectives” through contraceptive use may increase perceived relevance of family planning for AGYW. Furthermore, participants often discussed the contraceptive benefits of ideal birth spacing, inspiring inquiry into the ways contraceptive information is framed for young people. Human rights-based approaches to education have suggested similar framing around the perceived benefits, like achieving objectives and autonomy over pregnancy, to improve AGYW family planning engagement [53, 59]. Generally, targeted messaging approaches have had varying levels of effectiveness because they have not adopted a holistic or multi-level approach to addressing social norms [52, 56]. Therefore, holistic, sustained, high dosage educational efforts should be paired with tailored messaging using accessible platforms for AGYW, such as radio and television announcements and person-to-person information campaigns (social networks) [52, 55, 56, 59]. Ultimately, a multi-tiered approach that sensitizes AGYW family planning use, reduces misinformation through CSE, and improves relevance of sexual and reproductive health education through targeted messaging is recommended to reduce sociocultural barriers to desired family planning services and contraceptive information for AGYWLWH in Moshi, Tanzania.

Despite predominantly receiving contraceptive information from non-clinical sources pre-pregnancy, similar to adolescents in other LMICs, participants implied that they perceived clinicians as the most appropriate and trusted source of contraceptive information, paralleling preferences of AGYW from other LMICs about sources of contraceptive information [62]. As a result of their awareness of multiple methods, most participants recognized that possible side effects were individualized experiences. While this perceived individualization fostered uncertainty about method choice and use, it also promoted interest in individualized method choice and counseling, both which facilitate informed choice [59, 63]. As a result, participants’ desires for individualized choice motivated pursuit of family planning services post-pregnancy, where individual method counseling from a clinician facilitated desired use, aligning with rights-based recommendations to improve the quality of family planning programs [59, 63].

Given the preference for receiving contraceptive information from a trusted clinical source, it was unexpected that few individuals received family planning information from their HIV clinic, particularly because there have been efforts to integrate family planning services into routine HIV care with the support of the Tanzanian government [64]. Integrated family planning services have increased use of hormonal contraception and reduced unmet family planning need [52, 65, 66]. Since only two people sought family planning information from their HIV clinic, this data calls into question whether integration has been effective in this context, either because implementation did not reach these participants (i.e. not fully integrated) or did not work for these participants (i.e. participants did not think the information was relevant). Therefore, an evaluation of the implementation of integrated family planning services within the Kilimanjaro region would promote informed choice and human-rights centric care for AGYWLWH [52, 57, 59, 63].

Almost all participants thought hormonal contraception was important, emphasizing the autonomy and choice that came with use, regardless of whether they had the opportunity to experience family planning services. While participants had varied perceptions of and experiences with contraception and family planning services, many individuals recommended that AGYWLWH use contraception to prevent unintended pregnancies, meet their objectives, and plan for their desired family structure, implying a greater need for reproductive choice among AGYWLWH. Informed choice remains the gold standard for reproductive health care delivery [57, 63]. Unfortunately, a lack of informed choice for adolescent contraceptive use has been observed in other parts of Tanzania [42] and has been associated with long-acting reversible contraceptive (LARC) use in the Arusha region [61]. These experiences conflict with recommendations offered by Bruce’s “Fundamental elements of the quality of care” for family planning and informed choice [63], and therefore, likely act as a barrier to engagement with clinical family planning services. Additional inquiry into the experiences of AGYWLWH with family planning services is recommended to better understand desired patient and provider support in these conversations. Calls for increased provider training specific to adolescent sexual and reproductive health provision have become more prevalent [46, 52, 56, 58, 67], and implementation guidelines for adolescent responsive contraceptive services (ARCS) can be used to develop safe and welcoming environments for young people to receive family planning services with non-stigmatizing provider relationships [52, 56, 57, 59, 60].

### Limitations

While this participant population was a purposive sample, many AGYWLWH from the original SYV pilot study were no longer reachable, limiting the sample size. Similarly, only PE participants were able to join for a FGD; no NPE participants were able to join for a FGD. Despite this limitation, FGD results corroborated themes from NPE interviews, demonstrating thematic saturation and agreement among these participants with different experiences. Furthermore, the language used to describe the topic of family planning in Swahili has varied translations and understandings. Based on guidance from Tanzanian community advisory board members, the terminology “njia za uzazi wa mpango” was used, which means “reproductive planning methods” because these individuals suggested that “njia za uzazi wa mpango” was a more accessible phrase compared to others.

## Conclusion

AGYWLHW faced sociocultural barriers to accessing family planning services prior to their first pregnancy, which precluded access to contraceptive information while reducing the perceived relevance of said information pre-pregnancy. As a result, participants favored non-hormonal contraception before their first pregnancy, increasing the probability of unintended pregnancy. Despite these barriers, almost all participants shared that family planning services and hormonal contraception supported their health and family composition goals post-pregnancy. Looking to future generations, participants recommended that AGYWLWH use family planning services to achieve personal and professional goals, and they called for increased education about contraceptive methods to improve reproductive choice for AGYWLWH.

## Supplementary Information

Supplementary Material 1.

Supplementary Material 2.

Supplementary Material 3.

Supplementary Material 4.

Supplementary Material 5.

Supplementary Material 6.

## Data Availability

Available data upon request includes the primary analytic memos that outline quotes and thematic analysis for participants’ family planning attitudes, experiences, and knowledge. Full transcripts are not publicly available as they contain more sensitive information, which synthesized could lead to participant identification.
