# Supplementary material for: “She cannot use these methods before she has at least one child”: a qualitative study on family planning attitudes, knowledge, and experiences among adolescent girls and young women living with HIV in Moshi, Tanzania
Source: Reprod Health. 2026 Aug 4;23:201. doi: 10.1186/s12978-026-02421-x (PMC13721370; doi:10.1186/s12978-026-02421-x)
Supplement: Supplementary file 1 [file 12978_2026_2421_MOESM1_ESM.docx]

**Supplemental Material 1:**

**Focus Group Discussion Question guide for SRH with AGYWLWH
Version 1.0**

| 1. **Moderator’s name** |  |
| --- | --- |
| 2. **FGD#** |  |
| 3. **Discussion date (mm/dd/yyyy)** | \|___\|___\|/\|___\|___\|/\|___\|___\|___\|___\| |
| 4. **Time discussion began (hh:mm)** | \|___\|___ \| : \|___\|___\| am/pm |
| 5. **Time discussion ended (hh:mm)** | \|___\|___\| : \|___\|___\| am/pm |

**Step 1:** Complete Q1—3 above before the group discussion.

**Step 2:** Introduce yourself at the beginning of the discussion.

**Step 3:** Thank participants for taking part in the discussions.

**Step 4:**	Read *Pre-Discussion: Information about the study* to the participants.

**Step 5:** Review *Discussion Ground Rules.* Ask if there are any other rules to add.

**Step 6:**	Turn on audio recorder. Document time discussion begins in Q4 above.

**Step 7:**	Facilitate discussion.

**Step 8:**	Thank the participant at the end of the discussion. Ask if there are any questions.

**Step 9:**	Document time discussion ended in Q5 above.

**Step 10:** Provide reimbursement and document appropriately.

**Pre-Discussion: Information about the study	[10 min]**

Welcome to our conversation today. I will moderate the discussion. This means I will ask questions and listen to all of you talk with one another. I will not participate in the conversation. I would like you to feel free to talk with one another.

Today’s discussion will focus on common perceptions about and support desired for pregnancy protection and family planning adolescent girls and young women living with HIV. We will also be discussing motivations for joining and staying in research studies.

There are no right or wrong answers to the questions I’ll ask. I am interested in hearing your opinions—you all have the best idea about these topics, so please share your candid thoughts. On occasion, I may direct the conversation to stay on topic, but I’ll try not to do that often.

Sometimes in these types of discussions, some people talk a lot and some people to say very little. It’s important for us to hear from all of you because you all have your unique opinions. So, if one of you is sharing a lot, I may ask you to let others talk. And if someone is not saying much, I may ask them their opinion of the topic.

Please know that we will not share the information you tell us with people outside of this research team. However, we cannot guarantee that other participants in this room might not share what is said. So, please do not share information that you are not comfortable other people knowing.

We also encourage all of you to keep the information shared by other people private. So, please do not share this information with people who are not currently in this room.

I am audio recording the discussion today. This is so I can remember everything that is said. Any names that you happen to share during the discussion will be removed from any written record of the conversation. The audio recording will be stored securely and eventually destroyed after we publish the study’s findings.

Please know that participating in this discussion is voluntary. You can choose not to answer a question or you can stop participating at any time.

You will receive 5,000 TSH and your bus fare for taking part in the discussion. I will give these out at the end of the discussion. The discussion will take about 3 hours, and we will take a short break in the middle.

Do you have any questions for me so far about the discussion?

**Discussion ground rules**

***[Note to moderator: Explain to the participants that these are ground rules that you’d like everyone to respect. After they are read aloud, ask the participants if they have any rules to add.]***

- Be respectful: You don’t need to agree with each other, but you must listen and be respectful
- Turn off or silence and ignore cellular phones
- Take turns: One person should speak at a time
- Speak up
- Speak to each other
- What is shared in the room, stays in the room

OK -- Let’s get started!

To begin let’s go around the round and state the name that you’d like us to call you during our discussion today – it doesn’t have to be your real name.

***[***Turn on the audio recorder now.***]***

**Section 1: Motivations to Join and Continue Attending Research Studies	[15 min]**

***Interviewer script:*** *I’d like to start by asking you all about adolescent girls’ and young women’s motivations to join research studies and continue attending research activities.*

1. Why would an AGYWLWH be motivated **to join** a research study?
   i. Have others heard similar things? Has anyone heard different reasons?
   b. Why would they be motivated **to continue** attending study activities?
   c. What **prevents** AGYWLWH from attending study activities?
   d. What, if anything, would **help** people your age stay engaged with research? Why?

**Section 2: Protection from Pregnancy and Family Planning Preferences of AGYWLWH [30 min]**

***Interviewer script:*** *Now I’d like to ask you about pregnancy protection and family planning preferences of adolescent girls and young women living with HIV as well as community perceptions of pregnancy protection for AGYWLWH.*

2. **Before becoming pregnant, do AGYWLWH want family planning methods? Why/why not?**
   a. After becoming pregnant, do AGYWLWH want family planning methods? Why/why not?
   b. Why would someone want to have a child before using family planning methods or services?
      i. Probe: Are there any times AGYW would want to use family planning methods or services before having a child?
   c. **What methods are AGYWLWH comfortable using? Which ones are they uncomfortable using? Why?**
   d. What are common questions or concerns AGYWLWH have about njia? About planning for a family?
      i. Probe about misconceptions
3. **What do people in your community think about AGYW who want to/use contraception?**
   a. Probe: What are some of the things partners say about AGYW who want to or use njia? Why?
      i. ...things that parents say...
      ii. …things that teachers say...
      iii. ...things that nurses say...
      iv. Is it different for AGYWLWH?

**RANK LISTING ACTIVITY 1 [45 min]**

*Now, I will ask you about some of the challenges or difficulties AGYWLWH face when trying to protect themselves from pregnancy or when they have an unplanned pregnancy. For these questions, I would like to write down some of your responses on this paper. This will help us with our next conversation piece.*

4. **What are some of the challenges/difficulties that AGYWLWH face when trying to protect themselves from pregnancy? Why?**
   a. Probe: Have you heard of anyone having challenges...
      i. Trying to get family planning services?
      ii. At family planning services?
      iii. Using a family planning method?
      iv. Do adolescent girls and young women experience stigma from seeking out these services? If so, what is an example of the stigma faced?
   b. Have others heard similar stories? Or different stories? How have you learned about these experiences?
5. **What is most helpful to AGYWLWH when trying to protect themselves from pregnancy? Why?**
   a. Probe: What are some of the good experiences you’ve heard about with family planning?
      i. What made this experience good? Are there other activities, people, etc who can make family planning a good experience?

*Thank you for your comments. Are there any other responses you think need to be added to these lists?*

*Thank you. Now I am going to hand out paper and pens/pencils. I would like you to please write down what you think are the 3 greatest challenges/difficulties from this list that AGYWLWH face when trying to protect themselves from pregnancy. If there is another challenge/difficulty AGYWLWH face when trying to protect themselves from pregnancy, please write it on your paper.*

*[Once all participants are done writing] Thank you everyone. Now, please write down what you think are the 3 most helpful factors from this list for AGYWLWH when trying to protect themselves from pregnancy. Again, if there is another helpful factor for AGYWLWH when trying to protect themselves from pregnancy, please write it on your paper.*

6. Can someone share what they wrote as the top 3 challenges/difficulties AGYWLWH face when trying to protect themselves from pregnancy? Why did you rank these as the top 3?
a. Does anyone have the same 3 challenges? Why?
b. Does anyone have 3 different challenges? Why?
7. Can someone share what they wrote as the top 3 most helpful factors for AGYWLWH when trying to protect themselves from pregnancy? Why did you rank these as the top 3?
a. Does anyone have the same 3 helpful factors? Why?
b. Does anyone have 3 different helpful factors? Why?

*Thank you all for your participation. Now we are going to have a 15 minute break to grab bites, soda, and check up on watato nyie wote. Please bring me your papers. Thank you very much.*

*~ Break for grabbing bites and soda, checking watoto, using the bathroom, etc ~*

**Section 3: Strategies to support AGYWLWH in Achieving Their Family Plans**

**RANK LISTING ACTIVITY 2 [45 min]**

*Now, I will ask you about some potential strategies that AGYWLWH use when trying to protect themselves from pregnancy and how teachers, caregivers, healthcare professionals, community members or other people can help them protect themselves from pregnancy (if they want to have a pregnancy when they are older). We will use the same process as last time--I will write down some of your responses here, and then I will pass out paper for you to write on.*

8. **For AGYWLWH who want family planning methods, what are good strategies to help them protect against pregnancy or get family planning methods? Why?**
9. **How can teachers, healthcare professionals, community members, caregivers/parents, or other people best support AGYWLWH in protecting themselves from pregnancy?**
   a. *Probe about: improving services that already exist*

*Thank you for your answers. Are there any other responses you think need to be added to these lists?*

*Thank you. Now I am going to hand out paper and pens/pencils. I would like you to write down the 3 most strategies from this list that AGYWLWH can take to protect themselves from pregnancy. If there is another helpful strategy AGYWLWH can take to protect themselves from pregnancy, please write it on your list.*

*[Once all participants are done writing] Thank you everyone. Now, please write down the 3 best strategies from this list that teachers, healthcare workers, or community members can do to help AGYWLWH protect themselves from pregnancy. Again, if you think there is another strategy to support AGYWLWH protect themselves from pregnancy, please write it on your list.*

10. Can someone share the top 3 strategies for AGYWLWH to protect against pregnancy or get family planning methods? Why did you rank these as the top 3?
c. Does anyone have the same 3 strategies? Why?
d. Does anyone have 3 different strategies? Why?
11. Can someone share the top 3 strategies teachers, healthcare workers, or community members can do to support AGYWLWH to protect against pregnancy or get family planning methods? Why did you rank these as the top 3?
e. Does anyone have the same 3 strategies? Why?
f. Does anyone have 3 different strategies? Why?
12. Do you have other recommendations to other AGYWLWH about best… Why?
    a. Pregnancy prevention practices?
    b. Family planning methods?
    c. Family planning services?

*Thank you all for your participation. We have two more questions to discuss. Before we do, Leonia will collect your papers. Please hand them to her as we move into the next section. Thank you very much.*

**Section 4: Improving Family Planning Services for AGYWLWH	[30 min]**

***Interviewer script:*** *Now we are going to move to our next questions about improving family planning services for AGYWLWH.*

13. **When should AGYWLWH start learning about... [Why? Agree/disagree?] [15 min]**
    a. Disclosure?
    b. Protection from pregnancy?
    c. Family planning methods?
    d. When do adolescent girls typically start talking to a healthcare provider about their sexual health (e.g. STI prevention, contraception, etc.)? Who is usually the first person they talk to about their sexual health?
    e. **To what extent do you think adolescent girls have awareness of family planning options?**
    f. Where are good places for AGYWLWH to learn about sexual health and protection from pregnancy?
       i. Probe: Where do they like to learn about sexual health and protection from pregnancy?
       ii. Probe: If a nurse gave presentations about pregnancy protection or njia at Teen Club, would that be helpful?
14. **What do you feel is missing from pregnancy protection support or family planning services for AGYWLWH [can be clinical or in the community]? Why?**
    a. Which do you think would be the easiest to add or change? Why?
    b. Which do you think would be most impactful to add or change? Why?

**Section 4: Closing [10 min]**

That was the last of my questions for you today.

15. Is there any other information you’d like to share about how to best support AGYWHLWH in protecting themselves from pregnancy or achieving their family planning goals?

Do you have any final thoughts, recommendations, or questions that you’d like to ask me or the group?

I want to sincerely than you all for your time and for sharing your thoughts. Please help yourself to any remaining refreshments.

Thank you very much!
