# Supplementary material for: “She cannot use these methods before she has at least one child”: a qualitative study on family planning attitudes, knowledge, and experiences among adolescent girls and young women living with HIV in Moshi, Tanzania
Source: Reprod Health. 2026 Aug 4;23:201. doi: 10.1186/s12978-026-02421-x (PMC13721370; doi:10.1186/s12978-026-02421-x)
Supplement: Supplementary file 2 [file 12978_2026_2421_MOESM2_ESM.docx]

**Supplemental Material 2:**

**Questions Guide for In-Depth Interviews with AGYWLWH**

**Version 1.6**

| 1. **Interviewer name** |  |
| --- | --- |
| 2. **Participant ID#** |  |
| 3. **Interview date (mm/dd/yyyy)** | \|___\|___\|/\|___\|___\|/\|___\|___\|___\|___\| |
| 4. **Participant agrees to audio record interview** | ☐ Yes  ☐ No |
| 5. **Time interview began (hh:mm)** | \|___\|___ \| : \|___\|___\| am/pm |
| 6. **Time interview ended (hh:mm)** | \|___\|___\| : \|___\|___\| am/pm |
| 7. **Participant wants a summary of the study findings** | ☐ Yes  ☐ No |

**Step 1:** Complete Q1—3 above before starting the interview.

**Step 2:** Introduce yourself at the beginning of the interview.

**Step 3:** Thank participant for taking part in the interview.

**Step 4:**	Read *Pre-Interview: Information about the study* to the participant.

**Step 5:** Ask for the participant’s permission to record the interview. Tick appropriate box in Q4 above.

**Step 6:**	Turn on audio recorder if permitted. Document time interview begins in Q5 above.

**Step 7:**	Conduct interview.

**Step 8:**	Thank the participant at the end of the interview. Ask if has any questions.

**Step 9:**	Document time interview ended in Q6 above.

**Step 10:** Provide reimbursement and document appropriately.

**Step 11:** Ask participants if they want a summary of the interview findings. Document response.

**Pre-Interview: Information about the study**

The goal of this interview is to learn more about your sexual and reproductive health desires and ways you’ve addressed your desires. These findings will help us identify additional support to help adolescent girls and young women living with HIV achieve their desired family planning outcomes.

There are no right or wrong answers to the questions I’ll ask, only opinions. Please feel free to share your candid thoughts. You are the expert here, and there is no one else we can ask to get the unique information that you can tell me about your beliefs and experiences.

If you agree, I would like to audio record the interview because I want to make sure I don’t miss any of your comments. If you don’t want the interview audio recorded, I will take detailed notes during the interview instead. The recording and transcript will be stored securely at Duke University and Kilimanjaro Christian Medical Centre and eventually destroyed after we publish the study’s findings. The transcript will only be shared with the co-investigators of this study. Some quotes from the transcript may be used in publication, based on the analysis of the data. No names will be included in the transcript or publications.

To ensure that your privacy is protected and that we can speak freely, I’d like to ask that we talk in a space that is quiet and/or private for you. I understand interruptions may happen during our conversation and that sometimes these can’t be avoided. If too many interruptions happen, I may ask that we reschedule our discussion to ensure the quality of our conversation.

Please know that participating in this interview is voluntary. You can choose not to answer a question, or you can stop participating at any time.

You will receive 5,000 TSH and bus fare reimbursement for taking part in the interview. The interview will take about 90 minutes.

Do you have any questions for me so far about the interview?

***[If yes, answer the participant’s questions.]***

Are you okay with our conversation being audio recorded?

***[If yes, begin audio recording now.]***

***[If no]*** That’s okay, I’ll take detailed notes as we talk.

OK -- Let’s get started.

**Section 1: Get to Know You**

***Interviewer script:*** *I’d like to start by asking you about yourself and a little about your life.*

1. What brings you joy in life? What do you find challenging?
   a. What does a typical day look like for you?
   b. Probe: What do you enjoy? What do you dislike?
2. Do you have any children?
   a. [If yes] How many and what are their ages?

| **Child #** | **Age of Child** | **HIV status** | **Nevirapine at Birth** | **ARVs up to Now** | **Testing** | **Results of Tests** |
| --- | --- | --- | --- | --- | --- | --- |
|  | *Date Of Birth: |  |  |  |  |  |
|  |  |  |  |  |  |  |
|  |  |  |  |  |  |  |
|  |  |  |  |  |  |  |
|  |  |  |  |  |  |  |

b. [If yes] What was your age when you became pregnant with your first child?
   i. Tell me what it was like to be pregnant at [stated age].
      1. Did you plan to get pregnant at [stated age]? Why/why not? At what age did you want to start having children?
      2. Probe: Who were you living with? [Did you tell your partner?] How did your partner react? Were you and your partner married? How did your family react? How did your friends react? Why do you think they reacted this way?
      3. Probe: How did other people in your community act toward you? What did people say about you? Why do you think people said this?
      4. How did you feel about being pregnant at [stated] age?
      5. Can you describe the support you received while pregnant? Did you feel it was sufficient? Why/why not?
   ii. [If more than 1 child] Were all of your other pregnancies planned?
   iii. How many children do you want?
   iv. How did being pregnant impact your adherence to HIV medication?
       1. Why/why didn’t it(they) impact your adherence to medication?
       2. How did you cope with that impact?
c. [If no children] Why not?

**Section 2: Contraception Experience & Knowledge**

***Interviewer script:*** *Now we’re going to move into questions about sexual and reproductive health. Here, let’s talk about your experiences with family planning and knowledge about contraceptives.*

**HEARD:** ADVICE, SERVICES, METHODS, IMPACT

3. Please tell me what you have heard about family planning. *[If the participant seems stuck, offer ideas such as: school, radio advertisement, friends, parents, etc.]*
   i. Probe: What **recommendations** have you received about timing for building a family (finding a partner, when to have children, etc) from friends, parents, school, etc.?
   ii. Probe: What have you heard about family planning **services**?
   iii. Probe: What did you hear about **ways** to plan for families?
   b. When did you first hear advice about family planning? From whom did you hear this? How did this information make you feel? What were your thoughts about family planning after hearing this?
      i. How did this information influence where you sought family planning advice?
      ii. How did this information influence whether you wanted family planning services?
      iii. How did this information influence where you sought family planning services?
           1. Probe: Where have you considered going? Why?
      iv. How did this information influence your decision to use/not use family planning methods? How did this information influence which option(s) you sought?
   c. Where else did you get advice or information about how to plan for a family?
      i. Which sources of information influenced your decisions most? Why?
      ii. What information was important to you? Why?

**FP METHOD USE:** WHICH METHODS, EXPERIENCE, FULFILLED DESIRES

4. Have you ever used any action or method to prevent pregnancy?
   i. [If no] Have you ever tried the calendar/rhythm method, condoms, pullout method, devices, or other methods?
      1. [If yes] Continue here
      2. [If no] Skip to IF NEVER USED FP METHOD
   b. How old were you when you first tried a method to prevent pregnancy?
      i. Tell me about that experience.
      ii. Probe: Which method or action did you use? Why did you use this method? How did you feel about that method?
      iii. What factors encouraged your decision to continue/discontinue this method?
           1. [If discontinued] Did you discontinue this method within a year of starting? Why/why not? Did you switch to a different method within a year of starting? Why/why not?
      iv. Did you know your HIV status at the time?
5. [**If** have used] Tell me about your most recent (current) experience with family planning methods.
   a. Probe: Which method were you using? Why were you using this type of contraception? [If different from first] Why did you switch methods? What do you like about this method? What do you dislike about this method? Do you plan to continue using this method? Why/why not?
   b. Probe: Did you experience any side effects with this method? If so, what were they?
   c. Did your partner know that you are using this? Why/why not?
   d. Did you encounter challenges getting/using [contraceptive mentioned]? If so, what were they? How did you overcome these challenges?
   e. What factors encouraged your decision to continue/discontinue this method?
      i. [If discontinued] Did you discontinue this method within a year of starting? Did you switch to a different method within a year of starting?
   f. What helped you access/use this contraceptive? Why did this help?
   g. [If an object] Where did you get this contraceptive? How was this experience?
   h. Did this method of contraception fulfill your family planning desires? If so, how so? If not, why not?
   i. Is there anything different you would want to address your family planning desires? If so, what? If not, why not?
6. Have you used any other family planning methods that we haven’t discussed (examples: condom, calendar/rhythym, pull out method, devices, etc)?
   a. If so, what was it? Why did you use it? How was that experience (side effects, challenges, discontinuation/switching, like/dislike)?
7. Can I ask you specifically about:

| **Contraception** | **Have you ever heard of [x]?** | **What have you heard about [x]?** | **From whom did you hear this?** | **Have you ever used [x]? [About how old were you?]** | **How was your experience using [x]?** | **What are your opinions about [x]? Why?** |
| --- | --- | --- | --- | --- | --- | --- |
| **Male condom** |  |  |  |  |  |  |
| **Female condom** |  |  |  |  |  |  |
| **Birth control pill** |  |  |  |  |  |  |
| **Intrauterine device (IUD)** |  |  |  |  |  |  |
| **Contraceptive injection** |  |  |  |  |  |  |
| **Contraceptive implant or patch** |  |  |  |  |  |  |
| **Caps or diaphragms** |  |  |  |  |  |  |
| **Vaginal ring** |  |  |  |  |  |  |

8. [If used different methods] What motivated you to switch methods?
9. Have you ever tried to get a family planning method but were denied one? Please tell me about that experience.
10. Did you ever want a family planning method but felt you couldn’t get one? Please tell me about that experience.
11. Who do you feel comfortable discussing contraception with? Why?
    a. When was the last time you discussed contraception with these people? Why?
    b. What did you talk about?
    c. Who do you want to discuss contraception with but feel like you can’t? Why do you feel you can’t talk to them about it?

**IF NEVER USED FP METHOD**

12. Why haven’t you used any action or method to prevent pregnancy?
    a. Probe: Have you wanted to get a family planning method? If so, why? If not, why not?
       i. [If so] Which method would you want? Why?
13. Have you ever tried to get a family planning method but were denied one? Please tell me about that experience.
14. Did you ever want a family planning method but felt you couldn’t get one? Please tell me about that experience.
15. What do you do instead to prevent pregnancies? Why?
    a. Probe: Have you had challenges getting family planning services?
       i. If so, what were those challenges?

*CONTINUE WITH CHART QUESTIONS

**FP SERVICE USE:** WHERE, EXPERIENCE, FULFILLED DESIRES

16. Have you ever received family planning services?
    a. Tell me about the first time you received family planning services. [that experience]
       i. How old were you when you first received family planning services? Where did you receive family planning services? Why did you get services there?
          1. Probe: Did you get this service before or after your first pregnancy?
             a. [If before pregnancy] Why? Did you want family planning counseling? Why/why not?

i. Probe: [If yes] Why did you want family planning services?

ii. [If no] What motivated you to get this service?

b. [If not before pregnancy] Why? Why didn’t you pursue family planning services before your first pregnancy?
b. **Walk** me through your first experience once you arrived at the service.
   i. Probe: Who offered the services? What did they say to you? How was your experience with the [person who offered the service]? Why?
      1. Probe: How did they act toward you? How did they make you feel?
   ii. What did you want to get from the service? What did you get from the service?
       1. Did you want to get a family planning method? Why/why not?
          a. [If so] Was this the first time you tried to get contraception? Why/why not?
       2. Did you leave with a family planning method? Why/why not?
          a. [If yes] What family planning method did you get? Why? Did they explain side effects and proper use? How did this impact your sexual experience? Why?

i. Was this the method you wanted? Why/why not?

1. How easy or challenging was it to get the method you wanted? Please explain why it was [easy/challenging].

2. [If no] What contraceptive method did you want? Why?

3. [If no] What other challenges did you have in getting the contraceptive method you wanted? How did you overcome those challenges?

b. [If no] Did you get a family planning method somewhere else after the visit? Why/why not?

i. [If yes] Where did you go? Why did you go there?

iii. What other information did you want to learn? Were you able to ask all of your questions?
     1. Were your desires addressed? Why/why not?
     2. Do you think they were good at advising you? Why/why not?
iv. Have you returned since? Why/why not?
    1. Would you recommend this service to a friend? Why/why not?
c. How did this experience influence your thoughts about where and from whom to get contraception?
d. What were your opinions about family planning counseling before using this service? Why?
   i. Did your opinions change after receiving family planning counseling? If so, how? If not, why not?
e. Have you ever received clinical family planning services? Why/why not? How was this experience?

17. Have you had other family planning experiences that we haven’t discussed?
    a. If so, where? How were these experiences? Did you leave with your desires addressed? Why/why not?
    b. Have you had any uncomfortable experiences getting family planning counseling? Please tell me what happened.
    c. Have you received advice about planning pregnancies from other sources? If so, from whom? What did they say?
18. When you have gone to the CTC, did you ever talk about contraception with a doctor or nurse?
    a. [If yes] Why? What did they say? What happened next? Where did they refer you to? Did you go? Why/why not?
    b. [If not] Why not? Who else have you asked about contraceptives? What challenges did you face? Why?
19. Thinking about your experiences, what did you like about family planning counseling? Why?
    a. What didn’t you like from family planning counseling? Why?
    b. Where do you like to get family planning services? Why?
       i. Are there places you don’t like to go or places you avoid getting family planning services? Why?
    c. What would you like to see changed or added to family planning services? Why?
       i. Probe: Why is this important to you? Why would this be helpful?
       ii. How would you recommend the change(s) be implemented? Why?
       iii. What are the most important aspects to consider when changing/adding this? Why?
20. What has been most challenging for you in getting family planning services? Why? How have you overcome those challenges?
21. What has helped you get family planning services? Why did this help?

**IF NEVER RECEIVED FP SERVICES**

22. Why haven’t you received family planning services?
    a. Probe: Have you wanted to get family planning services? If so, why? If not, why not?
       i. [If so] Where would you want to go? Why? Where would you not want to go? Why?
       ii. [If so] What services would you want? Why?
    b. Probe: What are your opinions about family planning services? Why?
    c. Probe: Have you had challenges accessing family planning services?
       i. If so, what were those challenges?
    d. Have you looked for advice about planning pregnancies from another source? If so, which sources? Why?
23. When you have gone to the CTC, did you ever talk about contraception with a doctor or nurse?
    a. [If yes] Why? What did they say? What happened next? Where did they refer you to? Did you go? Why/why not?
    b. [If not] Why not? Who else have you asked about contraceptives?
    c. What challenges did you face? Why?

**Section 3: Relationship Influence on FP/Contraceptive Experience Health/IPV**

***Interviewer script:*** *Let’s now talk about your sense of autonomy in current and previous relationships.*

24. Tell me about the relationship you have/had with the partner of your first pregnancy.
    a. Probe: Are they your current partner? Did they know your HIV status? Why/why not?
    b. Before you had your first pregnancy, did you feel comfortable discussing contraception with this partner? Why/why not?
       i. Probe: What did they say to make you feel this way?
       ii. Probe: What did they do to make you feel this way?
       iii. How did this impact your use of/comfortability using contraception?
            1. Probe: Did you use contraception? If yes:
               a. Probe: Did your partner know about it? Why/why not?
               b. Probe: How did this impact your continued use of contraception?
       iv. [If not] How did you overcome this challenge? Did you use contraception with this partner?
    c. Do you feel your preferences about sex were respected? Why/why not?
       i. Probe: What did they say to make you feel this way?
       ii. Probe: What did they do to make you feel this way?
25. Do you feel comfortable discussing contraception with your current partner? Why/why not?
    a. Probe: What do they say to make you feel this way?
    b. Probe: What do they do to make you feel this way?
    c. How does this impact your use of/comfortability using contraception?
    d. [If not] How do you navigate this challenge (so you can use contraception)?
    e. How does this impact your ability to get the family planning services you desire? Why?
    f. Does your current partner know about your HIV status? Why/why not?

**Section 4: Abortion**

***Interviewer script:*** *Now I’d like to ask you about your opinions about abortion. As a reminder, if you’re uncomfortable with any question, you can say you don’t want to answer it, and we will move onto the next question.*

26. What are your opinions about abortion?
    a. Why? What makes you feel this way?
27. What are community perceptions about abortion?
    a. What do people say about abortion? What do people say about women who get abortions?
    b. How do people act to make you think this? How do people treat women who get abortions?
28. What are perceptions about abortion among other women your age in your community?
    a. What do people say to make you think this? How do people act to make you think this?
    b. Do you know someone who has tried to get an abortion?
       i. [If so] How did that make you feel? Why?
    c. If someone your age wanted to get an abortion, what challenges would they face?
       i. Why would they face these challenges?
       ii. What are some of their reasons for getting an abortion?
       iii. How do women your age abort their pregnancy?
       iv. Do you know of anyone who had a complication with their abortion? Why did they have this complication? What happened?
    d. Have you ever tried to get an abortion?
       i. [If yes] Please tell me about this experience.
          1. What are the reasons you wanted an abortion?
          2. How did you learn about where to get an abortion?
          3. Did you tell anyone? If yes, who did you tell? Why?
          4. What challenges did you face? Why?
          5. Probe: Did you experience any complications after the abortion? If so, what were they? How did you address these complications?
       ii. [If no] Would you feel comfortable seeking an abortion? Why/why not?
           1. Did you ever consider getting an abortion? Why/why not?

**Section 5: Closing**

That’s the end of the questions that I have for you today.

29. Is there any other information you’d like to share about your contraceptive or family planning desires?
30. Do you have any other recommendations for supporting adolescent girls or young women living with HIV in addressing their contraceptive or family planning desires?

I want to sincerely thank you for your time and for the helpful information that you provided. Would you like a summary of the study findings?

Thank you very much.
