# Supplementary material for: “She cannot use these methods before she has at least one child”: a qualitative study on family planning attitudes, knowledge, and experiences among adolescent girls and young women living with HIV in Moshi, Tanzania
Source: Reprod Health. 2026 Aug 4;23:201. doi: 10.1186/s12978-026-02421-x (PMC13721370; doi:10.1186/s12978-026-02421-x)
Supplement: Supplementary file 3 [file 12978_2026_2421_MOESM3_ESM.docx]

**Supplemental Material 3:**

**Questions Guide for In-Depth Interviews with AGYWLWH**

**Version 1.6 [non-pregnant]**

| 1. **Interviewer name** |  |
| --- | --- |
| 2. **Participant ID#** |  |
| 3. **Interview date (mm/dd/yyyy)** | \|___\|___\|/\|___\|___\|/\|___\|___\|___\|___\| |
| 4. **Participant agrees to audio record interview** | ☐ Yes  ☐ No |
| 5. **Time interview began (hh:mm)** | \|___\|___ \| : \|___\|___\| am/pm |
| 6. **Time interview ended (hh:mm)** | \|___\|___\| : \|___\|___\| am/pm |
| 7. **Participant wants a summary of the study findings** | ☐ Yes  ☐ No |

**Step 1:** Complete Q1—3 above before starting the interview.

**Step 2:** Introduce yourself at the beginning of the interview.

**Step 3:** Thank participant for taking part in the interview.

**Step 4:**	Read *Pre-Interview: Information about the study* to the participant.

**Step 5:** Ask for the participant’s permission to record the interview. Tick appropriate box in Q4 above.

**Step 6:**	Turn on audio recorder if permitted. Document time interview begins in Q5 above.

**Step 7:**	Conduct interview.

**Step 8:**	Thank the participant at the end of the interview. Ask if has any questions.

**Step 9:**	Document time interview ended in Q6 above.

**Step 10:** Provide reimbursement and document appropriately.

**Step 11:** Ask participants if they want a summary of the interview findings. Document response.

**Pre-Interview: Information about the study**

The goal of this interview is to learn more about your sexual and reproductive health desires and ways you’ve addressed your desires. These findings will help us identify additional support to help adolescent girls and young women living with HIV achieve their desired family planning outcomes.

There are no right or wrong answers to the questions I’ll ask, only opinions. Please feel free to share your candid thoughts. You are the expert here, and there is no one else we can ask to get the unique information that you can tell me about your beliefs and experiences.

If you agree, I would like to audio record the interview because I want to make sure I don’t miss any of your comments. If you don’t want the interview audio recorded, I will take detailed notes during the interview instead. The recording and transcript will be stored securely at Duke University and Kilimanjaro Christian Medical Centre and eventually destroyed after we publish the study’s findings. The transcript will only be shared with the co-investigators of this study. Some quotes from the transcript may be used in publication, based on the analysis of the data. No names will be included in the transcript or publications.

To ensure that your privacy is protected and that we can speak freely, I’d like to ask that we talk in a space that is quiet and/or private for you. I understand interruptions may happen during our conversation and that sometimes these can’t be avoided. If too many interruptions happen, I may ask that we reschedule our discussion to ensure the quality of our conversation.

Please know that participating in this interview is voluntary. You can choose not to answer a question, or you can stop participating at any time.

You will receive 5,000 TSH and bus fare reimbursement for taking part in the interview. The interview will take about 90 minutes.

Do you have any questions for me so far about the interview?

***[If yes, answer the participant’s questions.]***

Are you okay with our conversation being audio recorded?

***[If yes, begin audio recording now.]***

***[If no]*** That’s okay, I’ll take detailed notes as we talk.

OK -- Let’s get started.

**Section 1: Get to Know You**

***Interviewer script:*** *I’d like to start by asking you about yourself and a little about your life.*

1. What brings you joy in life? What do you find challenging?
   a. What does a typical day look like for you?
   b. Probe: What do you enjoy? What do you dislike?
2. Do you have any children?
   a. [If yes] SWITCH TO PREGNANT GUIDE
   b. [If no children] Would you like to have children?
      i. Why?/Why not?
      ii. How many would you like?
      iii. Do you have a desired timeline for your life/having children?
           1. Why do you want to follow this timeline?
           2. How did you come up with this timeline? (probe: about external)
      iv. What will you do to follow this timeline? (or *Do you have a plan for this timeline?*)

**Section 2: Contraception Experience & Knowledge**

***Interviewer script:*** *Now we’re going to move into questions about sexual and reproductive health. Here, let’s talk about your experiences with family planning and knowledge about contraceptives.*

ADVICE

3. **Please tell me what you have heard about family planning. *[If the participant seems stuck, offer ideas such as: school, radio advertisement, friends, parents, etc.]***
   i. Probe: What **recommendations** have you received about timing for building a family (finding a partner, when to have children, etc) from friends, parents, school, etc.?
   ii. Probe: What have you heard about family planning **services**?
   b. **When did you first hear advice about family planning?**
      i. What advice did they give?
      ii. From whom/where did you hear this?
      iii. How did this information influence your interest in family planning methods?
      iv. How did this information influence your decision to use/not use family planning methods?
      v. How did this information influence which option(s) you sought?
   c. **Where else have you received advice or information about how to plan for a family?**
      i. Which sources of information were most impactful to you? [Whose advice was most important to you?] Why? (Why did you use the information from x source to inform your family planning)
      ii. What information was important to you? Why?

**FP METHOD USE:** WHICH METHODS, EXPERIENCE, FULFILLED DESIRES

4. **What are your thoughts or opinions about family planning methods?**
   a. Why?
   b. What have you heard about family planning methods? *[If the participant seems stuck, offer ideas such as: good/bad methods, side effects, friends’ experiences, etc.]*
   c. Do you have any concerns about family planning methods? If so, what are they?
   d. What about family planning methods would be helpful to you?
   e. What do your friends say or think about family planning methods?
   f. What do your family members say or think about family planning methods?
5. **Who do you feel comfortable discussing contraception with? Why?**
   a. When was the last time you discussed contraception with them? Why?
   b. What did you talk about?
   c. Is there someone (else) you wish you could talk about contraception with? Why/why not?
6. **Have you ever used any action or method to prevent pregnancy?**
   i. [If no] Have you ever tried the calendar/rhythm method, condoms, pullout method, devices, or other methods?
      1. [If no] Skip to IF NEVER USED FP METHOD
   b. **[If yes] How old were you when you first tried a method to prevent pregnancy?**
      i. **Tell me about that experience.**
         1. Probe: Which method or action did you use? Why did you use this method?
         2. Probe: Did you like this method? Why/why not?
      ii. Why did you continue/discontinue this method?
          1. [If discontinued] Did you discontinue this method within a year of starting? Why/why not? Did you switch to a different method within a year of starting? Why/why not?
      iii. Did you know your HIV status when you first tried a method to prevent pregnancy?
7. **[If yes] Tell me about your most recent (current) experience with family planning methods.**
   a. Probe: Which method were you using? Why were you using this type of contraception?
      i. [If different from first] Why did you switch methods?
      ii. **What do you like about this method? What do you dislike about this method? Do you plan to continue using this method? Why/why not?**
   b. Probe: Did you experience any side effects with this method? If so, what were they?
   c. Did your partner know that you are using this? Why/why not?
   d. **Did you have challenges using [contraceptive mentioned]?**
      i. [If so] What were they? How did you overcome these challenges?
      ii. **Did you have challenges getting [contraceptive mentioned]?**
      iii. [If so] What were they? How did you overcome these challenges?
      iv. Do you know other people who had challenges getting family planning methods? What were those challenges?
   e. **Why did you continue/discontinue this method? Do you plan to continue using this method? Why/why not?**
      i. [If discontinued] Did you discontinue this method within a year of starting? Did you switch to a different method within a year of starting?
   f. **What helped you access/use this contraceptive? Why did this help?**
   g. **Where did you get this contraceptive? How was this experience?**
   h. **Did this method of contraception fulfill your family planning/pregnancy prevention desires?**
      i. If so, how so? If not, why not?
   i. **Is there anything different you would want to address your family planning desires?**
      i. If so, what? If not, why not?
8. **Have you used any other family planning methods that we haven’t discussed (examples: condom, calendar/rhythm, pull out method, devices, etc)?**
   a. If so, what was it? Why did you use it? How was that experience (side effects, challenges, discontinuation/switching, like/dislike)?
9. **Have you ever tried to get a family planning method but were denied one? Please tell me about that experience.**
10. **Did you ever want a family planning method but felt you couldn’t get one? Please tell me about that experience.**

**IF NEVER USED FP METHOD**

11. **Have you wanted to get a family planning method?**
    i. If so, why? If not, why not?
    ii. [If so] Which method would you want? Why?
    b. **[If so] What has prevented you from getting the family planning method? Have you had challenges getting family planning methods (getting approval from your parents or partner, receiving the method you wanted from a nurse)?**
       i. [If so] What were those challenges?
       ii. [If so] How have you overcome those challenges?
       iii. Do you know other people who had challenges getting family planning methods they wanted? What were those challenges?
12. **Have you ever tried to get a family planning method but were denied one?**
    a. [If yes] Please tell me about that experience.
    b. [If not] Why haven’t you tried to get a family planning method?
13. **Did you ever want a family planning method but felt you couldn’t get one?**
    a. [If yes] Please tell me about that experience.
    b. [If no] Why didn’t you want a family planning method?
14. What do you do instead to prevent pregnancies? Why?
    a. [If doesn’t take action] Probe: Why haven’t you used any action or method to prevent pregnancy?

**CONTRACEPTIVE KNOWLEDGE (all participants):**

15. **Can I ask you specifically about:**

| **Contraception** | **Have you ever heard of [x]?** | **What have you heard about [x]?** | From whom did you hear this? | **Have you ever used [x]?** [About how old were you?] | How was your experience using [x]? | **What are your opinions about [x]? Why?** |
| --- | --- | --- | --- | --- | --- | --- |
| **Male condom** |  |  |  |  |  |  |
| **Female condom** |  |  |  |  |  |  |
| **Birth control pill** |  |  |  |  |  |  |
| **Intrauterine device (IUD)** |  |  |  |  |  |  |
| **Contraceptive injection** |  |  |  |  |  |  |
| **Contraceptive implant or patch** |  |  |  |  |  |  |
| **Caps or diaphragms** |  |  |  |  |  |  |
| **Vaginal ring** |  |  |  |  |  |  |

a. [If used more than 1 method] Were you using any of these methods at the same time?
b. [If used different methods] What motivated you to switch methods?

**FP SERVICE USE:** WHERE, EXPERIENCE, FULFILLED DESIRES

16. **Please tell me, where are places you can get family planning advice?**
    a. **Of these places, which ones would you want to get family planning advice from?**
       i. Methods from?
       ii. Kwa nini? *[If participant is struggling, you can probe about what they’ve heard from other people or if they have had a bad experience.]*
    b. **Are there any places you wouldn’t want to go for family planning advice? For methods?**
    c. What are your opinions about family planning services? Why?
17. **Have you ever gone to family planning services?**
    a. [If no] Have you ever gone somewhere for family planning advice? Have you ever asked anyone for advice about how to plan for a family? *[If participant says they asked a nurse, doctor, pharmacist for advice, please continue here.]*
       i. [If no] Continue to IF NEVER RECEIVED FP SERVICES
    b. **[If yes] Tell me about your experience when you first received family planning services.**
       i. How old were you when you first went someplace for family planning services?
       ii. Where did you go for family planning services?
       iii. Why did you go there?
    c. **Walk me through your experience once you arrived at the family planning place.**
       i. Probe: Who offered the services? What did they say to you? How was your experience with the [person who offered the service]? Why?
          1. **Probe: How did they act toward you? How did they make you feel?**
       ii. **What did you want to get from the service? What did you get from the service?**
           1. Did you want to get a family planning method? Why/why not?
              a. [If so] Was this the first time you tried to get contraception? Why/why not?
           2. Did you leave with a family planning method? Why/why not?
              a. [If yes] What family planning method did you get? Why? Did they explain side effects and proper use? How did this impact your sexual experience? Why?

i. **Was this the method you wanted? Why/why not?**

1. How easy or challenging was it to get the method you wanted? Please explain why it was [easy/challenging].

2. [If no] What contraceptive method did you want? Why?

3. [If no] What other challenges did you have in getting the contraceptive method you wanted? How did you overcome those challenges?

b. [If no] Did you get a family planning method somewhere else after the visit? Why/why not?

i. [If yes] Where did you go? Why did you go there?

iii. What other information did you want to learn?
     1. **What questions did you have? Were you able to ask all of your questions?**
     2. **What questions do you still have about family planning services? Methods? Timelines?**
     3. Did you get everything you hoped to get from the encounter? Why/why not?
     4. Do you think they were good at advising you? Why/why not?
iv. Have you returned since? Why/why not?
    1. **Would you recommend this service to a friend? Why/why not?**
d. **How did this experience influence your thoughts about where and from whom to get contraception?**
e. What were your opinions about family planning counseling before using this service? Why?
   i. Did your opinions change after receiving family planning counseling? If so, how? If not, why not?

18. **Have you had other family planning experiences that we haven’t discussed?**
    a. If so, where? How were these experiences? Did you leave with your desires addressed? Why/why not?
    b. **Have you had any uncomfortable experiences getting family planning counseling? Please tell me what happened.**
    c. Have you received advice about planning pregnancies from other sources? If so, from whom? What did they say?
19. **When you have gone to the CTC, did you ever talk about contraception with a doctor or nurse?**
    a. [If yes] Why? What did they say? What happened next? Where did they refer you to? Did you go? Why/why not?
    b. [If not] Why not? Who else have you asked about contraceptives? What challenges did you face? Why?
20. **Thinking about your experiences, what did you like about family planning counseling? Why?**
    a. What didn’t you like from family planning counseling? Why?
    b. **What would you like to see changed or added to family planning services? Why?**
       i. Probe: Why is this important to you? Why would this be helpful?
       ii. How would you recommend the change(s) be implemented? Why?
       iii. What are the most important aspects to consider when changing/adding this? Why?
21. **What has been most challenging for you in getting family planning services? Why? How have you overcome those challenges?**
22. **What has helped you get family planning services? Why did this help?**
23. Do you know other people who had challenges getting family planning services? What were those challenges?
24. **Overall, what are your thoughts or opinions about family planning services?**
    a. Why?
    b. What have you heard about family planning services? *[If the participant seems stuck, offer ideas such as: good/bad places to get services, how nurses/doctors act, friends’ experiences, etc.]*
    c. What concerns do you have about family planning services?
    d. What do your friends say or think about family planning services?
    e. What do your family members say or think about family planning services?

**IF NEVER RECEIVED FP SERVICES**

25. **Why haven’t you asked for advice about family planning? (or, Why did you ask for advice from [person mentioned])?**
    a. Please tell me about the time you asked for advice about family planning.
       i. Probe: What did they say to you? How did they make you feel?
       ii. **What questions did you have about family planning?**
       iii. **What questions do you continue to have about family planning methods? Services? Timelines?**
            1. *[If comment they don’t have questions because they aren’t sexually active]* If you did have a partner or were sexually active, what questions would you have about family planning (methods, services, timelines)?
    b. Have you had any uncomfortable experiences trying to get advice about family planning? Please tell me what happened.
    c. Have you looked for advice about planning pregnancies from another source? If so, which sources? Why?
26. **Why haven’t you gone somewhere for family planning advice? Services?**
    a. Have you wanted to go somewhere for family planning services? If so, why? If not, why not?
       i. [If so] What services would you want? Why?
       ii. What services wouldn’t you want? Why?
    b. Have you had challenges accessing family planning services?
       i. If so, what were those challenges?
27. **When you have gone to the CTC, did you ever talk about contraception with a doctor or nurse?**
    a. [If yes] Why? What did they say? What happened next? Where did they refer you to? Did you go? Why/why not?
    b. [If not] Why not? Who else have you asked about contraceptives?
    c. What challenges did you face? Why?
28. **Have you had challenges getting family planning services (going to the location, getting a method from a nurse)? Why?**
    a. [If so] What were those challenges?
    b. [If so] How did you overcome those challenges?
    c. Do you know other people who had challenges getting family planning services? What were those challenges?
29. **What would you like to see changed or added to family planning services? Why?**
    a. Probe: Why is this important to you? Why would this be helpful?
    b. How would you recommend the change(s) be implemented? Why?
    c. What are the most important aspects to consider when changing/adding this? Why?
30. **Overall, what are your thoughts or opinions about family planning services?**
    a. Why?
    b. What have you heard about family planning services? *[If the participant seems stuck, offer ideas such as: good/bad places to get services, how nurses/doctors act, friends’ experiences, etc.]*
    c. What concerns do you have about family planning services?
    d. What do your friends say or think about family planning services?
    e. What do your family members say or think about family planning services?

**Section 3: Relationship Influence on FP/Contraceptive Experience Health/IPV**

***Interviewer script:*** *Let’s now talk about your sense of autonomy in current and previous relationships.*

31. Have you ever been in a romantic relationship?
    a. [If yes] Are you currently in a romantic relationship?
       i. [if no] Have you ever been sexually active?
          1. [If yes]: continue here
          2. [If no] Continue to NEVER SEXUALLY ACTIVE
32. **Tell me about the relationship you have/had with your current/past partner.**
    a. Are you happy in the relationship? Do you feel they respect you?
    b. Have you been sexually active with your partner?
       i. [If no] Have you ever been sexually active?
          1. *[If yes] continue here but about past partner*
          2. [If no] Why have you chosen not to engage in sexual activity? *[After answering this question, move to NEVER SEXUALLY ACTIVE]*
       ii. Did they know your HIV status? Why/why not?
       iii. Do you feel comfortable discussing contraception with your partner? Why/why not?
            1. Probe: What did they say to make you feel this way?
            2. Probe: What did they do to make you feel this way?
       iv. Do/did you use contraception with this partner (calendar method, condom, pill, etc)? Why/why not?
           1. Probe: Did your partner know about it? Why/why not?
    c. Do you feel your preferences about sex were respected? Why/why not?
       i. Probe: What did they say to make you feel this way?
       ii. Probe: What did they do to make you feel this way?

**NEVER SEXUALLY ACTIVE**

33. If you were in a relationship, would that influence your interest in contraceptives or family planning services? Why/why not?
    a. If you were sexually, would that influence your interest in contraceptives or family planning services? Why/why note?
34. If you were sexually active with your partner, would you talk to them about family planning? Why/why not?
    a. When would you feel comfortable talking to them about family planning? Why?

**Section 4: Abortion**

***Interviewer script:*** *Now I’d like to ask you about your opinions about abortion. As a reminder, if you’re uncomfortable with any question, you can say you don’t want to answer it, and we will move onto the next question.*

35. **What are your opinions about abortion?**
    a. Why? What makes you feel this way?
36. What are community perceptions about abortion?
    a. **What do people say about abortion? What do people say about women who get abortions?**
    b. **How do people act to make you think this? How do people treat women who get abortions?**
37. **How do other women your age in your community feel about abortion?**
    a. What do people say to make you think this? How do people act to make you think this?
    b. **Do you know someone who has tried to get an abortion? Please tell me about this.**
       i. [If so] How did that make you feel? Why?
    c. **If someone your age wanted to get an abortion, what challenges would they face?**
       i. Why would they face these challenges?
       ii. **What are some of their reasons for getting an abortion?**
       iii. **How do women your age abort their pregnancy?**
       iv. Do you know of anyone who had a complication with their abortion? Why did they have this complication? What happened?
    d. **Have you ever tried to get an abortion?**
       i. **[If yes] Please tell me about this experience.**
          1. What are the reasons you wanted an abortion?
          2. How did you learn about where to get an abortion?
          3. Did you tell anyone? If yes, who did you tell? Why?
          4. What challenges did you face? Why?
          5. Probe: Did you experience any complications after the abortion? If so, what were they? How did you address these complications?
       ii. **[If no] Would you feel comfortable seeking an abortion? Why/why not?**
           1. Did you ever consider getting an abortion? Why/why not?

**Section 5: Closing**

That’s the end of the questions that I have for you today.

38. Do you have any other thoughts about your family planning desires or interests?
39. Do you have other recommendations to help adolescent girls or young women living with HIV get family planning methods?

I want to sincerely thank you for your time and for the helpful information that you provided. Would you like a summary of the study findings?

Thank you very much.
