# Supplementary material for: “She cannot use these methods before she has at least one child”: a qualitative study on family planning attitudes, knowledge, and experiences among adolescent girls and young women living with HIV in Moshi, Tanzania
Source: Reprod Health. 2026 Aug 4;23:201. doi: 10.1186/s12978-026-02421-x (PMC13721370; doi:10.1186/s12978-026-02421-x)
Supplement: Supplementary file 4 [file 12978_2026_2421_MOESM5_ESM.docx]

**Supplemental Material 5. Diagram depicting participant eligibility and enrollment in the study.**
