# Supplementary material for: “She cannot use these methods before she has at least one child”: a qualitative study on family planning attitudes, knowledge, and experiences among adolescent girls and young women living with HIV in Moshi, Tanzania
Source: Reprod Health. 2026 Aug 4;23:201. doi: 10.1186/s12978-026-02421-x (PMC13721370; doi:10.1186/s12978-026-02421-x)
Supplement: Supplementary file 5 [file 12978_2026_2421_MOESM6_ESM.docx]

**Supplemental Material 6.** **Baseline comparison between all female SYV and current sub-study participants using Kruskal-Wallis and Fisher’s exact tests.**

 

 

Baseline characteristics between the original AGYWLWH from the SYV pilot

were compared to the sub-sample included in this qualitative study. These

samples were compared using Kruskal-Wallis tests to test differences in continuous

variables and Fisher’s exact tests to compare differences between proportions of

categorical variables. These methods were chosen due to the relatively small sample

size. While differences between these two cohorts may have emerged since baseline enrollment in the SYV pilot study, these limited differences at baseline demonstrate that this sub-study includes a representative sample of the AGYWLWH from SYV pilot cohort.
